# The European Union's responses to the COVID-19 crisis: How to fight a pandemic with the internal market

**DOI:** 10.1177/1023263X221130182

**Published:** 2022-08

**Authors:** Tomi Tuominen, Mirva Salminen, Kirsi-Maria Halonen

**Affiliations:** * University of Lapland, Rovaniemi, Finland; ** UiT – The Arctic University of Norway

**Keywords:** Security of supply, internal market, crisis, COVID-19, competence, legal base

## Abstract

In a liberalized market economy, states tend to purchase supplies required for producing publicly funded services, such as healthcare, from the markets instead of producing them themselves. The availability of critical supplies thus becomes a question of supply-side availability and supply chain management, and therefore their availability is conceptualized in terms of security of supply. The European Union's security of supply policy has focused on energy and security and defence. Security has primarily been sought from the markets, while the purpose of EU law has been to establish these markets and to guarantee their functioning. During the COVID-19 pandemic the European Union has sought to secure the availability of medical supplies by relying on a variety of internal market measures: free movement law, State aid law, competition law and public procurement law have all been used in this effort. Collectively these measures have aimed at securing the functioning of the markets and thus the availability of necessary supplies. Following the crisis, the European Union is now adopting a broader policy perspective to security of supply. However, this is still carried out mainly through internal market competences and by relying on the markets as the source of security.

## 1. Introduction

Once the severity of the COVID-19 pandemic became apparent to everyone, states rushed to acquire necessary medical supplies such as personal protective equipment (PPE), respirators and vaccines from wherever possible.^1^ Supply chain management, procurement rules and related issues instantly rose to the main headlines of all major news outlets.^2^ Although the term as such was not used that often, what the states were worried about was the *security of supply* of medical products that are critical when fighting a pandemic. In a liberalized market economy, supplies that are necessary for the production of publicly funded services are usually acquired through the markets instead of produced by the state. This goes both for services provided directly by the state and for services that the state has outsourced to private companies. The availability of critical supplies thus becomes a question of supply-side availability (and supply chain management), and therefore the issue is conceptualized in terms of security of supply.^3^ Even though healthcare is largely based on public funding and healthcare services are provided by public bodies, producing such services still requires medical supplies, which the state procures through the markets. Security of supply concerns are thus central during such a crisis as the COVID-19 pandemic.

The COVID-19 pandemic has received considerable attention in both public debates and academic discussions. This is for sure due to the severity of the crisis and the unprecedented nature of the responses that states around the world have resorted to in their attempts to manage the crisis. The preferable way to tackle the pandemic and the adequacy of the actions already taken have been studied extensively over the past three years. Most of the European legal scholarship on the pandemic has focused on sector specific issues and has taken place in specialist legal journals devoted to, for example, the regulation of health,^4^ public procurement,^5^ State aid^6^ or economic assistance to the Member States.^7^ However, an overall picture and assessment of the various measures that the European Union (EU) has taken to fight the COVID-19 pandemic in a medical capacity is missing.

The purpose of this article is to analyse the EU's efforts to tackle the COVID-19 pandemic in a medical capacity through the concept of security of supply and, furthermore, to analyse the EU's security of supply policy.^8^ The core of the article consists of a description and analysis of the measures that the EU has taken in order to fight the pandemic and recent policy initiatives and legislative proposals by the Commission that aim to increase the EU's crisis preparedness.^9^ To this end, the article addresses the following two research questions: What type of a regulatory response has the EU adopted to fight the pandemic in a medical capacity? What is the EU's security of supply policy like and how is it changing as a result of the pandemic?

This article does not assess how well the EU has fared in its responses to the COVID-19 pandemic, as this is not primarily a legal question but mainly an epidemiological and political issue. Rather, it contributes to current discussions on security of supply, especially in the context of human health as sparked by the pandemic, and the more general discussions on the role of the internal market in European governance. As is explained in the next section, security of supply has, from both a policy and a research perspective, been mainly an energy-related issue. This article opens the discussion on security of supply to the broader field of general EU law by addressing it from an internal market perspective. Furthermore, the article's findings are interesting with regard to the continued discussion on the role of the internal market in European governance and the EU's competences.

The main findings of the article can be summarized in the following. The EU's approach to security of supply has been narrow, focusing mainly on the supply of energy. Relying heavily on internal market legal bases, the EU has sought to create European energy markets through which security is fostered. Conversely, human health has been an internal market issue that has not been conceptualized as security of supply (section 2). Similarly, the EU's responses to the COVID-19 pandemic have focused on securing the functioning of the internal market and thus the availability of critical medical supplies. All aspects of internal market regulation have been utilized to this end (section 3). While recent legislative proposals broaden the EU's ambit of security of supply, this is still done by relying on internal market legal bases and, consequently, the markets as the source of security. The EU is now beginning to take a stronger role in security of supply, but the modus operandi remains the same (section 4). It is not surprising that the EU has fought the COVID-19 pandemic through the internal market, given that the internal market is a policy area in which the EU has broad legislative capacity, and that security of supply concerns relate to the markets. Due to the market-related nature of security of supply, the EU has been able to craft a security of supply policy although security of supply as such is not amongst the EU's competences as listed in the EU Treaties – and despite the fact that according to Article 4(2) TEU national security remains the sole responsibility of each Member State. This observation gives rise to both appraisal and criticism (section 5).

## 2. Security of supply in the European Union

This section outlines the meaning of ‘security of supply’ and parallel concepts in EU law and policy. Furthermore, the use of different legal bases is highlighted as well as the market-oriented approach in producing security. These observations are summarized in Table 1.

**Table 1.** The role of security of supply and markets in different policy areas.

Policy area	Meaning or role of security of supply	Utilized legal base	Role of the markets or relation to the markets
Energy	Specific use of the term security of supply. Defined as the uninterrupted availability of affordable energy.	Strong reliance on Article 114 TFEU (internal market), while also Articles 194 (energy) and 192 (environment) TFEU are utilized.	Aims to establish European energy markets that together with State action produce security of supply.
Security and defence	The term security of supply is used, although its definition remains vague. Relates to the availability of military equipment and the special role that they play with regard to national security.	Article 114 TFEU (internal market) together with the public security exception of Article 346 TFEU is utilized.	Aims to establish a European defence market that creates security of supply.
CIP	The term ‘security of supply’ is not used as the object of the policy are not supplies as such but infrastructures that are linked to the production or supply of supplies.	Article 352 TFEU as a residual competence is used.	Aims to protect the functioning of critical infrastructures for the sake of the economy.
Human health	Security of supply not recognized as its own concern. Focuses on health and safety aspects.	Article 168 TFEU (human health) and Article 114 TFEU (internal market) are both utilized.	Aims to establish an internal market for medical products.

In the EU most if not all focus on security of supply is given to energy. For example, the legal instrument colloquially referred to as the Security of Supply Directive concerned electricity supply specifically.^10^ This Directive has recently been replaced by a regulation that, likewise, concerns only the energy sector yet is referred to as the Risk Preparedness Regulation.^11^ The same goes for the literature on ‘security of supply’. Indeed, a much-cited article states: ‘Energy security, the security of energy supplies or more shortly security of supply are used as synonyms both in this article and in other parts of literature.’^12^ While there is an apparent historical context – related to geopolitics, economics and idiosyncratic national preferences – behind such focus on energy,^13^ it is somewhat strange that energy is the dominant if not sole factor when it comes to security of supply policy in the EU.

The EU has no legally binding definition for security of supply. The main policy focus is on energy security, which is nowadays defined as the uninterrupted availability of affordable energy.^14^ According to Article 4(2)(i) TFEU, energy policy falls under shared competences. The Lisbon Treaty introduced a specific legal base for energy: Article 194 TFEU. According to Article 194 TFEU, in the context of the establishment and functioning of the internal market and with regard for the need to preserve and improve the environment, Union policy on energy shall aim, in a spirit of solidarity between Member States, to: (a) ensure the functioning of the energy market; (b) ensure security of energy supply in the Union; (c) promote energy efficiency and energy saving and the development of new and renewable forms of energy; and (d) promote the interconnection of energy networks. In order to pursue these objectives, Article 194 TFEU makes it possible to adopt legally binding measures through the ordinary legislative procedure.

As can be seen from the wording of Article 194 TFEU, the internal market plays a central role with regard to energy security. Before the adoption of Article 194 TFEU other legal bases were used for the regulation of energy security, and despite the energy specific legal base now being in existence, these other legal bases continue being used. The most often used ones are Article 114 TFEU (internal market) and Article 192 TFEU (environment).^15^ While the term security of supply is used in EU secondary legislation, this is almost exclusively in the context of energy security. This is the case with regard to legislative instruments adopted on the basis of Article 194 TFEU,^16^ Article 122 TFEU (energy),^17^ Article 192 TFEU^18^ or Article 114 TFEU.^19^

The Oil Stocks Directive, adopted on the basis of Article 122 TFEU (then Article 100 TEC), stands out in contrast to this market-based approach.^20^ The Directive obliges the Member States to hold a reserve supply of corresponding to 90 days’ consumption. This is the only legislative instrument by the EU that sets such security of supply obligations on the Member States.^21^

Initially, the government-centred model of energy monopolies also guaranteed security of energy supply. As this model of producing security of supply was not seen as cost-effective, and along with the general trend of market liberalization, the emphasis shifted towards market-forces. Thus, the liberalization of European energy markets coincided with the change in the rationale for achieving security of supply in the context of energy. The emphasis did not shift just to the markets; rather, the emphasis shifted to creating European energy markets (as opposed to national markets).^22^ It is important to notice that before the liberalization agenda the Member States were in charge of producing security of supply, while now it is the EU (the Commission) that leads this process.^23^ As can be seen from the above-cited EU secondary law instruments, security of supply concerns are now tackled with both legislative instruments aiming at creating an internal market for energy and those aimed explicitly at creating security of supply.

The Common Security and Defence Policy (CSDP) is the other policy area in which the term ‘security of supply’ is used. Directive 2009/81/EC on defence and security procurement, for example, recognizes that ‘security of supply’ is a central concern in relation to defence and security equipment.^24^ Although the Directive does not seem to contain an explicit definition of security of supply, it is evidently linked to the conditions on which such equipment are procured, the way sub-contractors are used and supply chains managed.^25^ A similar observation can be made from Directive 2009/43/EC, which establishes a European license system for the transfer of defence-related products.^26^

Both Directive 2009/81/EC and Directive 2009/43/EC have been adopted on the basis of internal market competences.^27^ Thus, although not formally part of the CSDP, their existence is nevertheless clearly linked to it. As is explained in recital 2 of Directive 2009/81/EC: ‘The gradual establishment of a European defence equipment market is essential for strengthening the European Defence Technological and Industrial Base and developing the military capabilities required to implement the European Security and Defence Policy.’ We can thus conclude, that also within the scope of the CSDP, markets play a central role in producing security of supply.^28^

A third policy area that is clearly linked to security of supply concerns, although in which the term is not specifically used, is that of critical infrastructure protection (CIP). The central legal instrument in this policy area is Directive 2008/114/EC,^29^ adopted on the basis of Article 308 EC (now Article 352 TFEU), the so-called catch-all competence clause of the Treaties. The Directive establishes a procedure for the identification and designation of European critical infrastructures (ECIs), and a common approach to the assessment of the need to improve the protection of such infrastructures in order to contribute to the protection of people.^30^ Article 2 of the Directive defines as a critical infrastructure

an asset, system or part thereof located in Member States which is essential for the maintenance of vital societal functions, health, safety, security, economic or social well-being of people, and the disruption or destruction of which would have a significant impact in a Member State as a result of the failure to maintain those functions.

One of the objectives of CIP is to ensure the ‘stability of the internal market’ since ‘damage or loss of a piece of infrastructure in one [Member State] may have negative effects on several others and on the European economy as a whole’.^31^

On the basis of the above overview, it can be concluded that in the EU security of supply is mainly an energy related concern. While security of supply concerns are discernible from other policy contexts, in these other policy fields the issues are not framed in a similar manner through the concept of security of supply. The EU has utilized the internal market legal bases for adopting legislative acts in both energy and security and defence policy. Although CIP policy is not based on an internal market competence, one objective of this policy, too, is to secure the functioning of the internal market. Overall, the markets have a central role in these policy areas: either security is sought from the markets or markets are to be protected for the sake of security.

Lastly, a short remark on the EU's regulatory approach with regard to human health, as this policy area is central for the topic of this article. Medical devices have been regulated by the EU since 1990. The approach within this policy area, as well, has relied heavily on internal market legal bases. The adopted legal instruments have aimed to secure both market access and a high level of health and safety.^32^ All three of the central directives have been adopted on the basis of Article 100a EEC (now Article 114 TFEU): Directive 90/385/EEC,^33^ Directive 93/42/EEC^34^ and Directive 98/79/EC.^35^ Although a separate legal base for the regulation of human health was introduced in Maastricht, Article 152 EC (now Article 168 TFEU), it has not been used that often due to its limited scope. As stated in paragraph 5 of Article 168 TFEU, ‘any harmonisation of the laws and regulations of the Member States’ is prohibited, while paragraph 7 continues that the ‘Union action shall respect the responsibilities of the Member States for the definition of their health policy and for the organization and delivery of health services and medical care’.^36^ The narrow scope of Article 168 TFEU, on the one hand, and the broad scope of Article 114 TFEU, on the other hand, have meant that the EU has relied heavily on the latter in regulating human health.^37^ The mentioned directives exist for the purpose of establishing an internal market for medical devices, but they do not take into consideration the security of supply concerns related to such products.

## 3. Internal market measures as responses to the COVID-19 crisis

As can be seen from the overview in the previous section, security of supply concerns have not been prominent in the EU's policy on human health but have figured in other policy areas. While there have been some measures aimed at creating the necessary capacity ex ante, due to the lack of long-term planning the majority of the measures that the EU has taken during the COVID-19 pandemic have been rather ad hoc, utilizing the readily available, general internal market competences. This section outlines what type of measures the EU has taken in its effort to respond to the crisis, specifically from the perspective of how to make sure that necessary medical supplies (e.g., PPE, respirators and vaccines) are available. Measures falling under four different categories are shortly described: free movement law, State aid law, competition law, and public procurement law. The role that the markets play in these measures or how these measures envisage the role of the markets is highlighted. These observations are summarized in Table 2.

**Table 2.** Approaches in the use of internal market measures to tackle the COVID-19 pandemic.

Category of internal market measure	Type of response	Role of the markets or relation to the markets
Free movement law	Amending the Medical Device Regulation (EU) 2017/745; ad hoc measure	Loosen technical requirements so as to increase availability of products.
State aid law	Issuing a Temporary Framework; ad hoc measure	Maintain supply chains in order to secure supply of products.
Competition law	Reintroducing the use of comfort letters; in-between an ad hoc and already existing measure	Increase supply through cooperation between undertakings.
Public procurement law	Reliance on existing flexibility in the current Public Procurement Directive 2014/24/EU and the previously established JPA; already existing measures	Make procurement procedures flexible so as to make the procurement procedure faster; changes how the demand-side of markets works but does not affect supply-side.

The Medical Device Regulation (EU) 2017/745 establishes a framework to ensure the smooth functioning of the internal market as regards medical devices covered by the Regulation, taking as a base a high level of protection of health for patients and users, and taking into account the small- and medium-sized enterprises (SMEs) that are active in this sector.^38^ At the same time, the Regulation sets high standards of quality and safety for medical devices in order to meet common safety concerns as regards such devices. The Regulation is based on Articles 114 and 168(4)(c) (high standards of quality and safety for medicinal products) TFEU. The requirements set by the Regulation were intended to come into force from 26 May 2020.^39^ However, the Regulation was amended in April 2020 so as to take into consideration the need for an increased availability of vitally important medical devices across the EU, and at the same time to guarantee patient health and safety until the new legislation becomes applicable.^40^ In essence, technical requirements, which also aim to create an internal market for such products, were relaxed so as to make possible the production and use of vitally important medical devices during the crisis.

Already at the very beginning of the COVID-19 crisis, State aid rules were ‘practically suspended’ to enable Member States to respond to the economic effects of the pandemic.^41^ This was done on the basis of a Temporary Framework, first issued by the Commission in March 2020.^42^ The sixth and most recent decision on prolonging the Temporary Framework was taken in November 2021.^43^

The Temporary Framework sets out the possibilities Member States have to ensure liquidity and access to finance for undertakings that face a sudden shortage in order to allow them to recover from the economic downturn.^44^ To be precise, it sets out the compatibility conditions that the Commission will apply in principle to the aid granted by Member States under Article 107(3)(b) TFEU. This means that the aid giving Member State has the duty to show that the State aid measures notified to the Commission under this Temporary Framework are necessary, appropriate and proportionate to remedy a serious disturbance in the economy of the Member State concerned and that all the conditions of this framework are fully respected. The need to give financial assistance to companies is simple: due to the restrictions (i.e., lockdown measures) adopted as part of the responses to the pandemic business will suffer. To avoid the negative consequences of mass bankruptcies and to thus aid the economy, Member States want to give financial assistance to undertakings. As is always the case with State aid measures, a delicate balance needs to be struck between how much aid is necessary and what type of aid can be given while still retaining the integrity of the internal market and limiting distortions to competition.^45^

How do such State aid measures relate to security of supply? Although in the Commission's communications there is no direct link between State aid and security of supply, the link between such aid and supply chains is evident. Indeed, this link between supply chains and State aid measures is mentioned as one of the main reason for adopting the Temporary Framework: the COVID-19 outbreak is a major shock to the Union's economy, following which there ‘is a supply shock resulting from the disruption of supply chains’.^46^ The economic effects of as well as the control measures taken as a result of the COVID-19 crisis affect global supply chains severely.^47^ Apparently, the Commission's logic is that disruptions to supply chains, which can result in the required medical supplies (e.g., PPE, respirators and vaccines) not being available during the crisis, can to a certain extent be mitigated by State aid to strategically important sectors or undertakings.

In practice, from the perspective of national authorities the link between State aid and security of supply is evident: in order to ensure the availability of products and services, it is important to secure the survival of strategically important suppliers and sufficient provision capacity of critical products and services.^48^ In such a crisis situation, especially local companies are seen as important to secure supplies at the national level.^49^ State aid can also be given to strategic partners, such as national airline companies,^50^ as the upkeep of functioning air-routes is a security of supply concern.^51^

In April 2020, the Commission issued a Temporary Framework for assessing antitrust issues related to business cooperation in response to situations of urgency stemming from the COVID-19 crisis.^52^ The purpose of the communication is to allow undertakings to cooperate with each other in a manner that is necessary in order to overcome the COVID-19 crisis, yet which might be forbidden under Article 101 TFEU in normal circumstances. According to the Temporary Framework, cooperation between undertakings is not forbidden as long as they do not

give rise to an enforcement priority for the Commission, to the extent that such measures would be: (i) designed and objectively necessary to actually increase output in the most efficient way to address or avoid a shortage of supply of essential products or services, such as those that are used to treat COVID-19 patients; (ii) temporary in nature (i.e. to be applied only as long there is a risk of shortage or in any event during the COVID-19 outbreak); and (iii) not exceeding what is strictly necessary to achieve the objective of addressing or avoiding the shortage of supply.^53^

A notable feature of the Temporary Framework is how it has in practice reintroduced the use of comfort letters in EU competition law.^54^ Comfort letters refer to a practice whereby undertakings can ex ante ask the Commission's opinion on whether their practices are acceptable under Article 101 TFEU. Such dialogue between antitrust authorities and undertakings has been seen as a preferential way for combatting this crisis but also as a way to develop competition law in the future.^55^ For example, in March 2021 the Commission issued a comfort letter allowing cooperation between undertakings in order to upscale the production of COVID-19 vaccines.^56^

Lastly, public procurement has, understandably, had a central role as part of the responses to the COVID-19 crisis as fighting such a crisis requires for public authorities to acquire medical supplies (e.g., PPE, respirators and vaccines).^57^

The EU's public procurement regime is designed to support long-term strategic purchasing activities of public authorities. Its features such as central purchasing units and other forms of joint purchasing, multi-provider framework agreements and dynamic purchasing systems contribute to the purpose of securing constant supply.^58^ Indeed, the purpose of joint purchasing and multi-provider agreements is to tackle the problems associated with non-professional public buyers and delivery failures of individual contractors.^59^ In addition to the nature of ensuring supply provision in general, the public procurement framework also contains rules for exceptional circumstances that can be activated during a crisis. For instance, Article 32(2)(c) of the Public Procurement Directive 2014/24/EU provides for the possibility to award a contract directly to an economic operator in the event of extreme urgency brought about by events unforeseeable by the contracting authority and not attributable to the contracting authority.^60^ Similarly, Article 72(1)(c) allows, under certain conditions, contract amendments in cases where the need for modification has been brought about by circumstances that a diligent contracting authority could not foresee.

Taking into consideration both the mechanisms for urgency procurement and amendments of existing contracts as well as the procurement structures of aggregated purchasing supported by the EU's public procurement rules, it has been suggested that the public procurement framework is as such already capable of securing supply and to prepare for crises.^61^ However, some scholars have argued that the criteria for recourse to Article 32(2)(c) are too narrow, and that the Commission's Communication from April 2020^62^ explaining how the said article functions in a state of crisis is incorrect.^63^

The European Joint Procurement Agreement (JPA) was initially established in 2014 with view to organizing the procurement of vaccines and medications in preparation for a pandemic.^64^ Along with the other measures adopted in the wake of the crisis, the JPA was one of the central tools for the Member States to procure critical supplies to fight the pandemic.^65^ The legal nature and legal basis of the JPA is a bit ambiguous. Although it seems that the JPA is based on Article 5 of Decision No 1082/2013/EU,^66^ according to the Commission this is not the case and the JPA is in fact a ‘budgetary implementing measure’ of said decision.^67^ Decision No 1082/2013/EU itself is based on Article 168(5) TFEU, which concerns the protection of human health. Be that as it may, the JPA explicitly concerns public procurement, so therefore it can be classified as an internal market measure.

The purpose of the JPA is to enable the Member States and the EU institutions to jointly purchase medical supplies. Thus, the JPA does not oblige the Member States to anything but rather its purpose is to make the procurement of medical supplies more effective. In practice, the JPA enables the Member States and the EU institutions to coordinate their procurement procedures.^68^ A key question regarding the use of the JPA in the fight against the COVID-19 crisis has been whether such actions affect the integrity of the internal market.^69^ Simply put, centralising procurement actions changes the way markets work (who buys and how from whom). Such centralisation can stir up the markets.

In the previous section it was concluded that internal market legal bases have been utilized in crafting policies aimed at security of supply in a variety of different policy areas. On the basis of the overview provided in this section, we can conclude that during the COVID-19 crisis the EU has utilized all four types of internal market measures (free movement law, State aid law, competition law, and public procurement law) in its pursuit of security of supply. These have been mainly ad hoc reactions to the crisis, although the existing framework of internal market measures contains elements of flexibility that proved useful during the crisis. Thus, not only is security of supply policy crafted on the basis of internal market legal bases but also pursued through the full array of internal market measures.

## 4. Towards a broader conceptualization of security of supply?

As a result of the COVID-19 pandemic, the EU is now starting to adopt a broader conceptualization of security of supply; that security of supply concerns are becoming evident also in other policy contexts than just energy and security and defence. The Council has, for example, emphasised the need to enhance the efficiency of public procurement in order to tackle future crises, and especially how to hence ensure security of supply.^70^ At the end of 2020, the Commission has issued several legislative proposals that relate, on the one hand, to the further development of CIP,^71^ and, on the other hand, to the establishment of a ‘European Health Union’.^72^ Although the scope of security of supply concerns are thus broadened, this is still carried out through internal market competences and by relying on the markets as the source of security. The content of these proposals is briefly discussed in this section and the role that the markets play in them or how they relate to the markets is summarized in Table 3.

**Table 3.** The role of the markets in the EU's broadened security of supply policy.

Policy area	Utilized legal base	Role of the markets or relation to the markets
Broadened CIP policy	Proposed new Directive based on Article 114 TFEU, although the content of CIP policy is vastly broadened.	Broadened CIP policy secures the good functioning of the internal market regarding services essential for the maintenance of vital societal functions or economic activities. It is not the critical infrastructures as such that are protected by the new Directive but the maintenance of vital societal functions or economic activities in the internal market.
New European Health Union	Two proposed Regulations based on Article 168(5) TFEU (combating serious cross-border threats to health). One proposed Regulation based on both Article 114 TFEU and Article 168(4)(c) TFEU (high standards of quality and safety for medicinal products).	By protecting human health, the economy also becomes protected. Monitoring of the markets (availability of products) increases crisis preparedness.

When it comes to the proposal for a Directive on the resilience of critical entities, which is essentially about CIP, the link between such infrastructures and the internal market is described well by the Commission, according to whom ‘[t]he livelihoods of European citizens and the good functioning of the internal market depend on the reliable provision of services fundamental for societal or economic activities in many different sectors’.^73^ Thus, with this Directive the Commission

intends to create an all-hazards framework to support Member States in ensuring that critical entities are able to prevent, resist, absorb and recover from disruptive incidents, no matter if they are caused by natural hazards, accidents, terrorism, insider threats, or public health emergencies like the one the world faces today.^74^

The current CIP Directive 2008/114/EC, already discussed above in section 2, was adopted on the basis of Article 308 EC (now Article 352 TFEU) and concerned only two sectors: energy and transport. The proposed new Directive would be considerably broader as it would cover ten different sectors: energy, transport, banking, financial market infrastructures, health, drinking water, waste water, digital infrastructure, public administration, and space. The Directive would be based on Article 114 TFEU. Thus, the content of CIP would be vastly broadened yet this would be done through an internal market legal base.

The content of Article 308 EC, which was the legal base of Directive 2008/114/EC, differs from its current formulation in Article 352 TFEU: Article 308 EC could be used to ‘attain, in the course of the operation of the common market, one of the objectives of the Community’ for which the Treaties did not provide a legal basis; conversely, Article 352 TFEU can be used to attain any objective set out in the Treaties and not just those related to the internal market. This change in the wording of Article 308 EC has been said to have reflected ‘the nature of the Union as no longer restricted to a narrow common market objective’.^75^ So, why is the broadening of CIP policy based on the internal market competence of Article 114 TFEU and not on Article 352 TFEU? Is reliance on the markets still at the centre of the EU's rationale towards CIP? Or is the reason the lower threshold for the use of Article 114 TFEU (qualified majority instead of unanimity)?

The purpose of the proposed European Health Union is to enable the Member States to prepare and respond together to health crises; to make sure that medical supplies are available, affordable and innovative; and to enable countries to work together to improve prevention, treatment and aftercare for diseases such as cancer. The legislative proposals issued by the Commission focus on three issues: strengthening coordination at the EU level when facing cross-border health threats; revising the mandates of the European Centre for Disease Prevention and Control and of the European Medicines Agency to provide stronger surveillance, scientific analysis and guidance before and during a crisis; and setting up a new EU agency for biomedical preparedness.^76^

Two of the proposed regulations comprising the European Health Union would be based on Article 168(5) TFEU (combating serious cross-border threats to health),^77^ while the proposed Regulation on a reinforced role for the European Medicines Agency would be based on both Article 114 TFEU and Article 168(4)(c) TFEU (high standards of quality and safety for medicinal products).^78^ While the two regulations that would be based on Article 168(5) TFEU do not directly relate to the internal market, they are indirectly related to it since when ‘our health is in danger, our economies are in danger’, as the Commission has explained.^79^ The proposed Regulation on a reinforced role for the European Medicines Agency is relevant from the perspective of the internal market in that the Regulation aims to establish a system for the monitoring of shortages of medicinal products. Furthermore, this way the Regulation also purports to contribute towards crisis preparedness.^80^ Thus, the proposed Regulation aims to secure the availability of medicinal supplies through the markets.

Overall, these observations show that while the EU is now adopting a broader understanding of security of supply, this broadening is still based on the internal market competence of Article 114 TFEU and on a reliance on the markets as the source of security. This seems to be in line with the observation, discussed above in section 2, that Article 114 TFEU has always played a central role with regard to the EU's health policy.^81^

## 5. Concluding remarks

This article sought to address the following two research questions: What type of a regulatory response has the EU adopted to fight the COVID-19 pandemic in a medical capacity? What is the EU's security of supply policy like and how is it changing as a result of the pandemic? With regard to the first question, we conclude that the EU has fought the pandemic through the internal market. As part of its responses to the crisis, the EU has both adopted measures on the basis of internal market legal bases as well as relied on the markets in its quest for the availability of necessary medical supplies. When it comes to the second question, we conclude that prior to the crisis the EU's security of supply policy has focused on energy and security and defence. In these two policy fields the internal market has had a central role: regulation has utilized internal market legal bases and security has been sought from the markets. Following the crisis, security of supply concerns are now broadened to encompass also other policy fields, even if the role of the markets remains the same.

What broader conclusions can be drawn from these answers? First, the explanation for this is rather obvious: given that the internal market is a policy area in which the EU has broad legislative capacity, and that security of supply concerns relate closely to the markets,^82^ it is perhaps not that surprising that the EU has fought the COVID-19 pandemic through the internal market. In other words, due to the market-related nature of security of supply, the EU has been able to craft a security of supply policy although security of supply as such is not amongst the EU's competences as listed in the EU Treaties – and despite the fact that according to Article 4(2) TEU national security remains the sole responsibility of each Member State. This observation gives rise to both appraisal and criticism. The positive aspect is that despite lacking a specific competence the EU has managed to take a variety of actions to support the Member States in tackling the COVID-19 pandemic. These efforts have been directed at both the human health-related aspects of the crisis as well as at those related to the economy and the markets. Indeed, often these intertwine, for which reason the rationale of protecting human health through market-based measures seems appropriate.

However, the actions taken by the EU, and the broader picture that they paint of the EU's competences and methods of functioning, also give rise to criticism or at least open up new questions that still need to be answered. The EU's competences are sectorally delineated in that it has competence in some policy areas (e.g., the internal market) while not in others (e.g., national security). Moreover, the EU's competences are functional (or purposive) in that they are to be used to attain specified objectives (e.g., the establishment and functioning of the internal market in the case of Article 114 TFEU).^83^ This means that the internal market is a policy area in which the scope of the EU's legislative competences are extremely broad and therefore the EU often regulates issues through internal market competences. Yet, whilst doing this a market logic is ingrained into the policy issue in question.^84^ The regulation of human health serves as an example.^85^ Such a market-based approach to the regulation of human health by the EU has been criticized for failing to consider the non-market values, such as solidarity and dignity, that are usually associated with human health.^86^ It has also been argued that the Court of Justice, as the interpreter of the appropriate use of legal bases, has ‘viewed and construed health matters on a market making or market enabling rationale rather than a social policy rationale’.^87^

In an analogous manner, we pose the question whether the market-related approach to security of supply has failed to adequately prepare the EU for a crisis such as the one presented by the COVID-19 pandemic. Does a market-based security of supply policy facilitate adequate preparation for and reaction to such crises, or would some other type of an approach to securing the availability of critical supplies and the continued functioning of public services (such as healthcare) suffice better? It needs to be recalled that during 2020 the main concern was not the lack of means to purchase required supplies but the lack of supply. Furthermore, although the EU comprises one internal market, in a situation such as the COVID-19 pandemic the Member States become competitors to each other within the internal market (and in addition to all other countries in the world for that matter) for the scarce supply of necessary medical supplies. Consequently, are the markets the best means for states to secure the availability of critical products? Is the EU, due to the nature of the competences conferred to it, destined to foster the security of supply solely through a market-based rationale?

Currently, security of supply still is a mainly national concern. The EU may use its internal market legal bases for pursuing security of supply related concerns, but only through a market-based manner; and it may support the functioning of thus established markets with measures based on State aid law, competition law and public procurement law. In other words, the EU tries to create the markets through which the Member States should take care of security of supply, yet the Member States are ultimately responsible for security of supply and must provide the necessary fallback if the markets fail to deliver. The alternative – to increase the EU's competences, perhaps by introduction other than functionally defined purposive competences – would require completely changing the modus operandi.

## Notes

1. See e.g. *Reuters*, ‘Exclusive: EU States Need 10 Times More Coronavirus Equipment – Internal Document’, 25 March 2020; *EUobserver*, ‘EU Fighting Shortages and Faulty Medical Supplies’, 2 April 2020.

2. See e.g. *Financial Times*, ‘Supply Chains Need Some Love During the Coronavirus Pandemic’, 8 April 2020; *Bloomberg News*, ‘Battered and Bruised, Supply Chains Shift to Recover-and-Survive Mode’, 26 April 2020.

3. On the definition of security of supply, see C. Egenhofer et al., ‘Market-Based Options for Security of Energy Supply’, *Fondazione Eni Enrico Mattei* (2004), Nota Di Lavoro 117.2004, https://www.ceps.eu/download/publication/?id=4911&pdf=1114.pdf.

4. See e.g. A. Alemanno (ed.), Taming COVID-19 By Regulation, special issue, 11 *European Journal of Risk Regulation* (2020), p. 187.

5. See e.g. A. Sanchez-Graells, ‘Procurement in the Time of COVID-19’, 71 *Northern Ireland Legal Quarterly* (2020), p. 81; L. Farca and D. Dragos, ‘Resilience in Times Of Pandemic: Is the Public Procurement Legal Framework Fit for Purpose?’, *Transylvanian Review of Administrative Sciences* (2020), p. 60; T. Kotsonis, ‘EU Procurement Legislation in the Time of COVID-19: Fit for Purpose?’, 19 *Public Procurement Law Review* (2020), p. 199; A. La Chimia, ‘Procuring for COVID-19: Housebound Reflections on a Procurement Earthquake’, 19 *Public Procurement Law Review* (2020), p. 161; G.L. Albano, ‘Homo Homini Lupus: On the Consequences of Buyers’ Miscoordination in Emergency Procurement for the COVID-19 Crisis in Italy’, 19 *Public Procurement Law Review* (2020), p. 213.

6. See e.g. G. De Stefano, ‘Covid-19 and EU Competition Law: Bring the Informal Guidance On’, 11 *Journal of European Competition Law & Practice* (2020), p. 121; C. Fumagalli, M. Motta and M. Peitz, ‘Which Role for State Aid and Merger Control During and After the Covid Crisis?’, 11 *Journal of European Competition Law & Practice* (2020), p. 294; P. Nicolaides, ‘Application of Article 107(2)(b) TFEU to Covid-19 Measures: State Aid to Make Good the Damage Caused by an Exceptional Occurrence’, 11 *Journal of European Competition Law & Practice* (2020), p. 238.

7. See e.g. D. Busch, ‘Is the European Union Going to Help us Overcome the COVID-19 Crisis?’, 15 *Capital Markets Law Journal* (2020), p. 347; T. Arnold, M. Gulati and U. Panizza, ‘How to Restructure Euro Area Sovereign Debt in the Era of Covid-19’, 15 *Capital Markets Law Journal* (2020), p. 322; B. de Witte, ‘The European Union's COVID-19 Recovery Plan: The Legal Engineering of an Economic Policy Shift’, 58 *Common Market Law Review* (2021), p. 635.

8. Thus, this article does not address the responses taken by the EU or the European Central Bank with regard to the adverse effects that the crisis has had on the European economy. See ibid.

9. For an updated timeline of EU action, see https://ec.europa.eu/info/live-work-travel-eu/coronavirus-response/timeline-eu-action_en.

10. Directive 2005/89/EC of the European Parliament and of the Council of 18 January 2006 concerning measures to safeguard security of electricity supply and infrastructure investment, [2006] OJ L 33/22.

11. Regulation (EU) 2019/941 of the European Parliament and of the Council of 5 June 2019 on risk-preparedness in the electricity sector and repealing Directive 2005/89/EC, [2019] OJ L 158/1.

12. C. Winzer, ‘Conceptualizing Energy Security’, 46 *Energy Policy* (2012), p. 36.

13. See K. Talus, *EU Energy Law and Policy: A Critical Account* (Oxford University Press, 2013), chapters 2 and 3; D.R. Helm, ‘European Energy Policy’, in E. Jones, A. Menon and S. Weatherill (eds.), *The Oxford Handbook of the European Union* (Oxford University Press, 2012), p. 556.

14. See K. Huhta, ‘Trust in the Invisible Hand? The Roles of the State and the Markets in EU Energy Law’, 13 *The Journal of World Energy Law & Business* (2020), p. 1.

15. See K. Huhta, ‘In Between State Sovereignty and Transnational Economic Integration: Article 194(2) TFEU and the Evolution of EU Competences in the Energy Sector’, 70 *International & Comparative Law Quarterly* (2021), p. 991.

16. E.g. Regulation (EU) 2019/943 of the European Parliament and of the Council of 5 June 2019 on the internal market for electricity, [2019] OJ L 158/54; Directive (EU) 2019/944 of the European Parliament and of the Council of 5 June 2019 on common rules for the internal market for electricity and amending Directive 2012/27/EU, [2019] OJ L 158/125; Regulation (EU) 2019/941.

17. Council Directive 2009/119/EC of 14 September 2009 imposing an obligation on Member States to maintain minimum stocks of crude oil and/or petroleum products, [2009] OJ L 265/9.

18. Directive 2009/28/EC of the European Parliament and of the Council of 23 April 2009 on the promotion of the use of energy from renewable sources and amending and subsequently repealing Directives 2001/77/EC and 2003/30/EC, [2009] OJ L 140/16; Regulation (EC) No 106/2008 of the European Parliament and of the Council of 15 January 2008 on a Community energy-efficiency labelling programme for office equipment (recast version), [2008] OJ L 39/1.

19. Directive 2009/72/EC of the European Parliament and of the Council of 13 July 2009 concerning common rules for the internal market in electricity and repealing Directive 2003/54/EC, [2009] OJ L 211/55; Regulation (EC) No 714/2009 of the European Parliament and of the Council of 13 July 2009 on conditions for access to the network for cross-border exchanges in electricity and repealing Regulation (EC) No 1228/2003, [2009] OJ L 211/15; Regulation (EC) No 713/2009 of the European Parliament and of the Council of 13 July 2009 establishing an Agency for the Cooperation of Energy Regulators, [2009] OJ L 211/1. These have now all been repealed by the legislative measures adopted on the basis of Article 194 TFEU.

20. Council Directive 2009/119/EC.

21. For the perspective of the Finnish national competent authority, see https://www.huoltovarmuuskeskus.fi/en/international-co-operation/eu-jurisdiction-in-security-of-supply-matters.

22. While there has recently been a slow trend back from a pure market-based approach to a government-based, interventionist approach, which also encompasses solidarity claims between the Member States, this is only so with regard to natural gas and the infrastructure necessitated by it but not with regard to electricity. See K. Huhta, 13 *The Journal of World Energy Law & Business* (2020), p. 2.

23. K. Talus, *EU Energy Law and Policy: A Critical Account*, p. 98 and 285; K Talus, *Introduction to EU Energy Law* (Oxford University Press, 2016), p. 1.

24. See Directive 2009/81/EC of the European Parliament and of the Council of 13 July 2009 on the coordination of procedures for the award of certain works contracts, supply contracts and service contracts by contracting authorities or entities in the fields of defence and security, and amending Directives 2004/17/EC and 2004/18/EC, [2009] OJ L 216/76, Recitals 8 and 9.

25. Ibid., Article 23.

26. Directive 2009/43/EC of the European Parliament and of the Council of 6 May 2009 simplifying terms and conditions of transfers of defence-related products within the Community, [2009] OJ L 146/1.

27. Directive 2009/43/EC is based on Article 95 EC (now Article 114 TFEU) whereas Directive 2009/81/EC is based on Articles 47(2) (now Article 53 TFEU) and 95 EC.

28. Further, see D. Keohane (ed.), *Towards a European Defence Market* (Chaillot Papers, No 113, November 2008).

29. Council Directive 2008/114/EC of 8 December 2008 on the identification and designation of European critical infrastructures and the assessment of the need to improve their protection, [2008] OJ L 345/75.

30. Ibid., Article 1.

31. Proposal for a Directive of the Council on the identification and designation of European Critical Infrastructure and the assessment of the need to improve their protection, COM(2006) 787, 3.

32. See M. Guy and W. Sauter, ‘The History and Scope of EU Health Law and Policy’, in T. Hervey, A. Young and L. Bishop (eds.), *Research Handbook on EU Health Law and Policy* (Edward Elgar Publishing, 2017), p. 26.

33. Council Directive 90/385/EEC of 20 June 1990 on the approximation of the laws of the Member States relating to active implantable medical devices, [1990] OJ L 189/17.

34. Council Directive 93/42/EEC of 14 June 1993 concerning medical devices, [1993] OJ L 169/1.

35. Directive 98/79/EC of the European Parliament and of the Council of 27 October 1998 on in vitro diagnostic medical devices, [1998] OJ L 331/1.

36. Further, see D.S. Martinsen, ‘Governing EU Health Law and Policy – On Governance and Legislative Politics’, in T. Hervey, A. Young and L. Bishop (eds.), *Research Handbook on EU Health Law and Policy*, p. 37.

37. See V. Delhomme, ‘Emancipating Health from the Internal Market: For a Stronger EU (Legislative) Competence in Public Health’, 11 *European Journal of Risk Regulation* (2020), p. 747.

38. Regulation (EU) 2017/745 of the European Parliament and of the Council of 5 April 2017 on medical devices, amending Directive 2001/83/EC, Regulation (EC) No 178/2002 and Regulation (EC) No 1223/2009 and repealing Council Directives 90/385/EEC and 93/42/EEC, [2017] OJ L 117/1.

39. Ibid., Article 1(2).

40. See Regulation (EU) 2020/561 of the European Parliament and of the Council of 23 April 2020 amending Regulation (EU) 2017/745 on medical devices, as regards the dates of application of certain of its provisions, [2020] OJ L 130/18.

41. See A. Biondi and O. Stefan, ‘EU Health Union and State Aid Policy: With Great(er) Power Comes Great Responsibility’, 11 *European Journal of Risk Regulation* (2020), p. 894.

42. Commission Communication, Temporary Framework for State aid measures to support the economy in the current COVID-19 outbreak, [2020] OJ C 91/1.

43. Commission Communication, Sixth Amendment to the Temporary Framework for State aid measures to support the economy in the current COVID-19 outbreak and amendment to the Annex to the Communication from the Commission to the Member States on the application of Articles 107 and 108 of the Treaty on the Functioning of the European Union to short-term export-credit insurance, [2021] OJ C 473/1.

44. Temporary Framework 2020, [2020] OJ C 91/1, para. 11.

45. Temporary Framework 2021, [2021] OJ C 34/6, para. 2.

46. See Temporary Framework 2020, [2020] OJ C 91/1, para. 1 and 2.

47. See D. Guan et al, ‘Global supply-chain effects of COVID-19 control measures’, 4 *Nature Human Behaviour* (2020), p. 577.

48. See P. Henty, ‘Procuring in a Pandemic: Cash-flow, Contracts, Construction, Cards and (Curtailing) Competition’, 19 *Public Procurement Law Review* (2020), p. 156; P. Henty, ‘Procuring in a Pandemic in the UK: A Review of the Public Procurement Policy Note “Responding to COVID-19”’, 19 *Public Procurement Law Review* (2020), p. 146.

49. See ‘Procurement Policy Note – Reserving Below Threshold Procurements’, Cabinet Office, Action Note PPN 11/20.

50. See ‘State aid: Commission approves around €350 million Finnish support to compensate Finnair for damages suffered due to coronavirus outbreak’, European Commission, Press release, 12 March 2021.

51. When giving State aid to Finnair, the national airline, the Finnish Government argued that the air-routes maintained by Finnair are crucial for Finland's security of supply. See ‘Government plans to support Finnair with EUR 600 million State guarantee’, Government Communications Department, Press Release 157, 20 March 2020.

52. See Commission Communication, ‘Temporary Framework for assessing antitrust issues related to business cooperation in response to situations of urgency stemming from the current COVID-19 outbreak’, [2020] OJ C 116/7.

53. Ibid., para. 15.

54. See J. Buhart and D. Henry, ‘COVID-20: The Comfort Letter Is Dead. Long Live the Comfort Letter?’, 43 *World Competition* (2020), p. 305.

55. G. De Stefano, 11 *Journal of European Competition Law & Practice* (2020), p. 121.

56. See ‘Comfort letter: cooperation at a Matchmaking Event – Towards COVID19 vaccines upscale production’, COMP/E-1/GV/BV/nb (2021/034137). Brussels, 25 March 2021.

57. See A. Sanchez-Graells, 71 *Northern Ireland Legal Quarterly* (2020), p. 81.

58. See e.g L. Farca and D. Dragos, *Transylvanian Review of Administrative Sciences* (2020), p. 60.

59. See e.g. C. Risvig Hamer, ‘Regular Purchases and Aggregated Procurement: The Changes in the New Public Procurement Directive Regarding Framework Agreements, Dynamic Purchasing Systems and Central Purchasing Bodies’, 13 *Public Procurement Law Review* (2014), p. 201; M. Andrecka, ‘Framework Agreements: Transparency in the Call-off Award Process’, 10 *European Procurement & Public Private Partnership Law Review* (2015), p. 231.

60. Directive 2014/24/EU of the European Parliament and of the Council of 26 February 2014 on public procurement and repealing Directive 2004/18/EC, [2014] OJ L 94/65.

61. See L. Farca and D. Dragos, *Transylvanian Review of Administrative Sciences* (2020), p. 60.

62. See Commission Communication, ‘Guidance from the European Commission on Using the Public Procurement Framework in the Emergency Situation Related to the COVID-19 Crisis’, [2020] OJ C 108/1.

63. See P Telles, ‘Extremely Urgent Public Procurement under Directive 2014/24/EU and the COVID-19 Pandemic’, 2 *Maastricht Journal of European and Comparative Law* (2022), p. 215.

64. Commission Decision COM(2014) 2258 final on approval of the Joint Procurement Agreement to procure medical countermeasures pursuant to Decision 1082/2013/EU, 10 April 2014. See N. Azzopardi-Muscat, P. Schroder-Back and H. Brand, ‘The European Union Joint Procurement Agreement for Cross-border Health Threats: What is the Potential for this New Mechanism of Health System Collaboration?’, 12 *Health Economics Policy and Law* (2017), p. 43.

65. See E. McEvoy and D. Ferri, ‘The Role of the Joint Procurement Agreement during the COVID-19 Pandemic: Assessing Its Usefulness and Discussing its Potential to Support a European Health Union’, 11 *European Journal of Risk Regulation* (2020), p. 851.

66. See Decision No 1082/2013/EU of the European Parliament and of the Council of 22 October 2013 on serious cross-border threats to health and repealing Decision No 2119/98/EC Text with EEA relevance, [2013] OJ L 293/1, Article 5.

67. See Commission, ‘Explanatory note on the Joint Procurement Agreement’, December 2015, section 2.4.

68. A Sanchez-Graells, 71 *Northern Ireland Legal Quarterly* (2020), p. 84.

69. See E. McEvoy and D. Ferri, 11 *European Journal of Risk Regulation* (2020), p. 851.

70. Conclusions of the Council meeting (25 November 2020), ‘Public Investment through Public Procurement: Sustainable Recovery and Reboosting of a Resilient EU Economy’, 7.

71. Proposal for a Directive of the European Parliament and of the Council on the resilience of critical entities, COM(2020) 829.

72. See Commission Communication, ‘Building a European Health Union: Reinforcing the EU's resilience for cross-border health threats’, COM(2020) 724; Commission proposal for a Regulation on a reinforced role for the European Medicines Agency in crisis preparedness and management for medicinal products and medical devices, COM(2020) 725; Commission proposal for a Regulation amending Regulation (EC) No 851/2004 establishing a European Centre for disease prevention and control, COM(2020) 726; Commission proposal for a Regulation on serious cross-border threats to health and repealing Decision No 1082/2013/EU, COM(2020) 727.

73. See ‘The Commission proposes a new directive to enhance the resilience of critical entities providing essential services in the EU’, Commission, News, 16 December 2020.

74. Ibid.

75. See A. Rosas and L. Armati, *EU Constitutional Law: An Introduction* (3rd edition, Hart, 2018), p. 25.

76. See https://ec.europa.eu/info/strategy/priorities-2019-2024/promoting-our-european-way-life/european-health-union_en.

77. Commission proposal COM(2020) 726.

78. Commission proposal COM(2020) 725.

79. See ‘Questions and Answers: Building a European Health Union: Stronger crisis preparedness and response for Europe’, Commission, Press release, 11 November 2020.

80. Commission proposal COM(2020) 725, Recitals 12 and 13.

81. See V. Delhomme, 11 *European Journal of Risk Regulation* (2020), p. 747.

82. At least within the current paradigm of how such security is to be produced.

83. See S. Weatherill, ‘Competence Creep and Competence Control’, 23 *Yearbook of European Law* (2004), p. 1; G. de Búrca, ‘The Constitutional Challenge of New Governance in the European Union’, 56 *Current Legal Problems* (2003), p. 403.

84. See G. Davies, ‘Democracy and Legitimacy in the Shadow of Purposive Competence’, 21 *European Law Journal* (2015), p. 2.

85. See V. Hatzopoulos, ‘Healthcare in the Internal Market’, in P. Koutrakos and J. Snell (eds.), *Research Handbook on the Law of the EU's Internal Market* (Edward Elgar Publishing, 2017), p. 166.

86. Ibid., 166.

87. V. Kosta, *Fundamental Rights in EU Internal Market Legislation* (Hart, 2014), p. 243.

